# Soluble Urokinase Plasminogen Activator Receptor (suPAR) Plasma Concentration Is Reduced Using Minimized Extracorporeal Circulation: Results of a Secondary Analysis of a Prospective Observational Study

**DOI:** 10.3390/jcm14145020

**Published:** 2025-07-16

**Authors:** Thomas S. Zajonz, Fabian Edinger, Juliane Götze, Melanie Markmann, Michael Sander, Christian Koch, Emmanuel Schneck

**Affiliations:** Department of Anesthesiology, Operative Intensive Care Medicine and Pain Therapy, University Hospital of Giessen and Marburg, Rudolf-Buchheim-Strasse 7, 35392 Giessen, Germany; thomas.s.zajonz@chiru.med.uni-giessen.de (T.S.Z.);

**Keywords:** biomarker, risk prediction, renal failure, adverse event, inflammation

## Abstract

**Background:** Minimized extracorporeal circulation (miECC) was developed to mitigate the adverse effects of cardiopulmonary bypass (CPB), yet its impact on soluble urokinase plasminogen activator receptor (suPAR) is unclear. SuPAR has been linked to adverse outcomes, including acute kidney injury (AKI). This study investigated perioperative suPAR kinetics in patients undergoing cardiac surgery with miECC or conventional CPB (cCPB) and explored its association with AKI, postoperative delirium (POD), and infections. **Methods**: This study is a secondary analysis of an observational cohort of 79 cardiac surgical patients. It evaluates perioperative suPAR levels and their association with the type of CPB used (miECC vs. cCPB) and postoperative adverse outcomes, including POD, AKI, and infections. Statistical analyses included repeated measures ANOVA, Wilcoxon tests, logistic regression, and ROC curve analysis to assess the predictive value of suPAR for these outcomes. **Results**: During surgery, suPAR significantly increased to higher levels with the use of cCPB compared to miECC (*p* = 0.027; odds ratio of 0.69 [0.57–0.84], *p* < 0.001). The use of miECC was an independent influencing factor on suPAR (−0.41 ± 0.1; *p* < 0.001). Regardless of the type of CPB, suPAR levels differed significantly between patients with and without kidney damage (*n* = 25; no AKI: 1.6 [1.1–2.0], AKI: 1.7 [1.3–2.4], *p* < 0.001). Multivariate regression analysis showed that AKI was an independent influencing factor on suPAR (−0.49 ± 0.1; *p* < 0.001). SuPAR demonstrated only low predictive value for AKI and could not predict POD. **Conclusions**: This study provides evidence that miECC is associated with lower intraoperative suPAR levels, suggesting a reduced inflammatory response compared to cCPB. While suPAR levels were significantly higher in patients with AKI, their predictive value for AKI remains limited. Furthermore, suPAR did not predict POD but was elevated in patients with pneumonia.

## 1. Background

Cardiopulmonary bypass (CPB) remains an essential technique in cardiac surgery and has undergone continuous optimization over the past decades to reduce its adverse effects on the inflammatory response, coagulation, and organ dysfunction, including acute kidney injury (AKI) and neurological complications [1,2,3]. To counteract these adverse effects, minimized extracorporeal circulation (miECC) systems have been introduced. These systems aim to reduce hemodilution, inflammatory activation, and oxidative stress by employing a closed circuit with lower priming volume and reduced foreign surface contact [3]. Despite these theoretical advantages, the impact of miECC on inflammatory biomarkers and clinical outcomes remains an area of ongoing investigation [2,4,5].

Soluble urokinase plasminogen activator receptor (suPAR) has emerged as a promising biomarker reflecting systemic inflammation and immune activation [6]. suPAR is derived from the cleavage of the membrane-bound urokinase plasminogen activator receptor (uPAR) and is involved in various pathological processes, including immune response modulation and tissue remodeling [7]. Elevated suPAR levels have been linked to worse clinical outcomes in a variety of conditions, including sepsis, chronic kidney disease, and cardiovascular diseases [6,7,8,9,10]. Importantly, suPAR has also been proposed as a predictor for AKI, as it reflects both acute and chronic inflammatory activity affecting renal function, particularly after cardiac surgery [8,11].

Given the role of systemic inflammation in postoperative complications, suPAR may also serve as a useful biomarker for other adverse outcomes in cardiac surgery patients. While elevated suPAR levels have already been linked to increased long-term mortality after cardiac surgery [12], their prognostic value for other adverse events, such as infections or postoperative delirium (POD), remains unclear. Furthermore, it has not yet been investigated whether the type of CPB system used during surgery affects suPAR kinetics and, consequently, its prognostic value.

In this study, we investigated the perioperative course of suPAR in patients undergoing cardiac surgery with either miECC or cCPB. The primary objective was to determine whether the use of miECC alters suPAR kinetics compared to cCPB. Additionally, we explored the association between suPAR levels and key postoperative complications, including AKI, delirium, and infections.

## 2. Methods

### 2.1. Study Design

This study presents a secondary analysis of an observational study including 100 cardiac surgical patients at the University Hospital of Giessen [13]. It was registered in the German Clinical Trials Register (trial registration: DRKS00010959) and performed with permission of the local ethics committee (Justus Liebig University Giessen, Giessen, Germany; approval number AZ: 30/16). Written informed consent was obtained from all patients. This study was conducted according to the principles of the Declaration of Helsinki [14]. The methods and results are presented according to the Strengthening the Reporting of Observational Studies in Epidemiology (STROBE) guidelines [15]. Patients were enrolled between September 2016 and January 2020.

### 2.2. Study Endpoints and Definition of Outcome Parameters

The primary objective of this study was to quantify perioperative suPAR levels and assess the potential impact of the type of CPB used, comparing miECC with cCPB.

The secondary objective was to evaluate the prognostic value of suPAR levels for the occurrence of POD, AKI, and postoperative infections (defined as pneumoniae, catheter-related bloodstream infections, and urogenital infections).

AKI was defined according to the Kidney Disease Improving Global *Outcome* initiative (KDIGO) [16].

A resident experienced in the applied delirium screening tests conducted the following assessments. Post-extubation patients were monitored for POD over seven days using the ICDSC and CAM-ICU [17,18]. A positive result in either screening tool was considered indicative of POD. Assessments were performed daily from postoperative day 1 to 7. To minimize bias, all examinations were conducted by a single investigator under the supervision of the principal investigator and attending intensive care physicians. The evaluations were based on ICD-10 criteria [19]. Sedation levels were assessed using the Richmond Agitation-Sedation Scale, and patients with a score of ≤−2 were excluded from testing and reassessed after four hours.

Infectious complications were identified through the screening of the inflammatory laboratory parameters, microbiological findings, and the attending physician’s documentation.

### 2.3. Patient Recruitment

Patients were recruited between September 2016 and January 2020. Patients were included if elective coronary artery bypass graft (CABG) surgery with postoperative intensive care treatment was performed. Further, inclusion criteria included age ≥ 18 years, elective on-pump CABG surgery, and the ability to communicate in German or English. Patients were excluded in case of missing consent, denial of participation, pregnancy, preoperative atrial fibrillation, severe bradycardia (<60 bpm; types: sinus bradycardia, atrial fibrillation with low frequency, nodal rhythm, and second- or third-degree atrioventricular block), acute infection before surgery, pre-existing autoimmune disease, immunomodulatory medication, left ventricular ejection fraction < 30%, and renal insufficiency (KDIGO score > 2). Further exclusion criteria were pseudocholinesterase deficiency, chronic cognitive dysfunction (e.g., history of schizophrenia, other severe psychiatric conditions, or dementia with inability to adequately answer the CAM-ICU or ICDSC), and recent or persistent neurological impairment (e.g., acute cerebral infarction, intracranial bleeding, or acute meningitis in the last three months prior to study inclusion leading to inability to answer the CAM-ICU or ICDSC).

### 2.4. Management of Cardiopulmonary Bypass

The patients in this non-interventional study underwent standard anesthetic induction with sufentanil (0.25–0.5 μg/kg), etomidate (0.1–0.2 mg/kg), and pancuronium (0.05–0.1 mg/kg). Anesthesia was maintained using propofol (3 mg/kg/h) and sufentanil (0.3–1 μg/kg/h). Central venous access was established via the internal jugular vein, and arterial blood pressure was monitored through the radial artery.

Both cCPB and MiECC utilized the S5 Heart-Lung Machine (LivaNova, London, UK) equipped with membrane oxygenators. MiECC employed the CAPIOX^®^ FX 15 Oxygenator (Terumo, Tokyo, Japan), while cCPB used the Inspire^®^ 6 F Oxygenator (LivaNova, London, UK) alongside a specialized perfusion tubing system (LivaNova, London, UK). For cCPB, a roller pump and an additional reservoir (LivaNova, London, UK) were implemented, whereas MiECC operated with a centrifugal pump, without the need for a reservoir or hemofilter.

**Figure 1:**
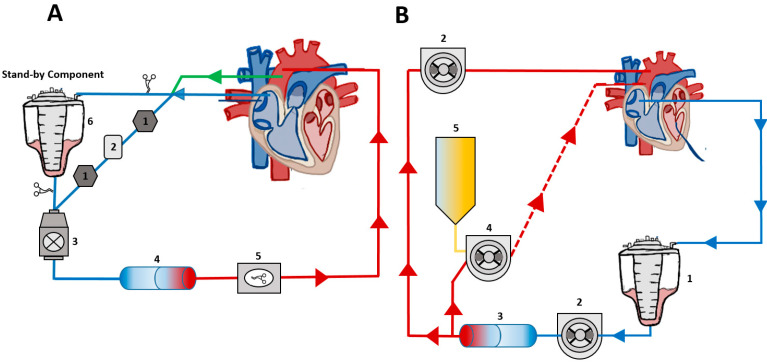
Diagrammatic representation of a minimized extracorporeal circulation (miECC) and a conventional cardiopulmonary bypass (cCPB), (**A**) (adapted from Anastasiadis et al. [20], 1: air detector, 2: air trap, 3: centrifugal pump, 4: oxygenator, 5: automatic arterial line clamp, 6: standby component (hard shell reservoir), green line: vent; (**B**) 1: hard shell reservoir, 2: double head roller pump, 3: oxygenator, 4: cardioplegia pump, 5: cardioplegia.

The priming solution for the circuits consisted of 1 L of crystalloid (Sterofundin Iso^®^, Braun, Melsungen, Germany), 250 mL of 15% mannitol (Seraq-Wiessner, Naila, Germany), and 50 mL of 20% albumin (CSL Behring, Marburg, Germany). For MiECC, 10,000 I.U. of heparin was added, while 2500 I.U. were required for cCPB. Details on the used CPB types are shown in Figure 1 and Supplemental Table S1.

### 2.5. Sample Processing

Blood was collected at six time points: immediately after induction of anesthesia (T1); 15 (T2) and 60 (T3) minutes after the commencement of CPB, respectively; and 15 (T4) and 120 (T5) minutes after the end of CPB after admission to the intensive care unit (ICU). Last, blood was drawn through the arterial line with the routine laboratory control in the early morning of the first postoperative day (T6). Blood was collected in ethylenediaminetetraacetic acid (EDTA) tubes (approximately 20 mL). Plasma samples were stored at −80 °C.

Clinical and laboratory data were obtained from the patient data management system (IMESO GmbH, Giessen, Germany).

### 2.6. Quantification of suPAR

The plasma concentration of suPAR was quantified using a human suPAR ELISA Kit (MyBiosource, Catalog No.: MBS7606253, San Diego, CA, USA) according to the manufacturer’s instructions.

### 2.7. Statistical Analysis

All numeric data were expressed as median and interquartile range (IQR). If summarized data are stated, the summarization occurred as follows: preoperative T1, intraoperative T2–T4 and postoperative T5–T6. The analysis of variations in plasma suPAR levels across different time points was performed by repeated measures ANOVA, followed by the Tukey HSD test. The comparison of operative suPAR levels for patients with POD or AKI versus those without, respectively, was conducted by the Wilcox Test. Categorical data have been compared using Fisher’s Exact Test. The influence of suPAR on pneumonia, POD, or AKI has been examined by logistic regression analysis, while receiver operating characteristic (ROC) curve analyses were used for the calculation of area under the ROC curve (AUCROC), sensitivity, and specificity of inflammatory and renal parameters. *p*-values < 0.05 were considered statistically significant. All data were stored in an external database (Microsoft Excel, Redmond, WA, USA). Data were analyzed using R version 4.3.2 (31 October 2023; www.r-project.org, accessed on 13 July 2025).

## 3. Results

### 3.1. Study Cohort

Of the 100 patients included in the primary study, sufficient sample volume for this secondary analysis was achievable for 79 patients. The characteristics of these 79 patients are shown in Table 1. Only two of 79 patients did not suffer from any pre-existing disease, which were spread evenly among the miECC and cCBP groups. Differences have been observed for a higher incidence of myocardial infarction within the 90 days prior to surgery (*p* < 0.01) and stroke (*p* < 0.05) in the cCPB group. There were no cases of acute cerebral infarction included in this study, and all strokes had occurred at least three months prior to surgery. In general, preoperative plasma troponin levels were low; however, they were significantly higher in patients undergoing cCPB (2.24 [1.2–2.9]) compared to those with miECC (0.21 [0.06–0.85]; *p* = 0.008). Association with suPAR concentrations occurred for myocardial infarction within 90 days (odds ratio: 1.31 [1.08–1.60]; *p* = 0.07). No patient died during the observational period.

### 3.2. Primary Endpoint Analysis

Overall, without consideration of time points, suPAR concentrations were significantly lower in patients undergoing MiECC compared to cCPB (*p* < 0.001). Detailed comparisons of the timepoints are shown in Figure 1A, while summarized data of the pre-, intra-, and postoperative time points are shown in Figure 1B (preoperative T1|intraoperative T2–T4|postoperative T5–T6, Table 2 and Table 3). Summarized data generally showed a significantly increased amount of suPAR during surgery and significantly decreased amounts postoperatively. During surgery, suPAR significantly increased to higher levels with the use of cCBP compared to miECC (*p* = 0.027, Figure 1B, logistic regression: odds ratio: 0.69 [0.57–0.84]; *p* < 0.001).

Multivariate regression analysis showed that MiECC was an influencing factor on suPAR independent of POD, pneumonia, myocard infarction, angina pectoris, and pre-existing diseases in general (−0.38 ± 0.1; *p* < 0.001).

### 3.3. Secondary Endpoint Analysis

#### 3.3.1. Acute Kidney Injury

**Figure 2:**
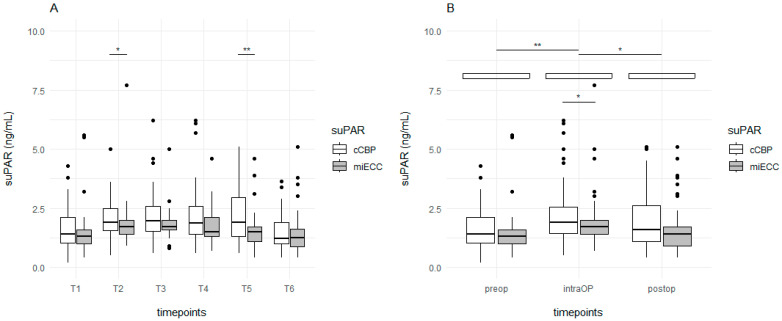
Boxplots showing the time course of suPAR in dependence of miECC and cCPB for single timepoints (**A**) and preoperative, operative, and postoperative periods (**B**). Overall, the level of suPAR is statistically significantly lower when using miECC as compared to cCBP (*p* < 0.001). SuPAR levels significantly increased during surgery (*p* = 0.008) and decreased again postoperatively (*p* = 0.04). With miECC, suPAR levels significantly increased less during OP than with cCBP (*p* = 0.03). Abbreviations: cCPB = conventional cardiopulmonary bypass; miECC = Minimized extracorporeal circulation; preOP = preoperative; postOP = postoperative; OP = intraoperative; suPAR = Soluble urokinase plasminogen activator receptor. * = *p* < 0.05; ** = *p* < 0.01.

**Figure 3:**
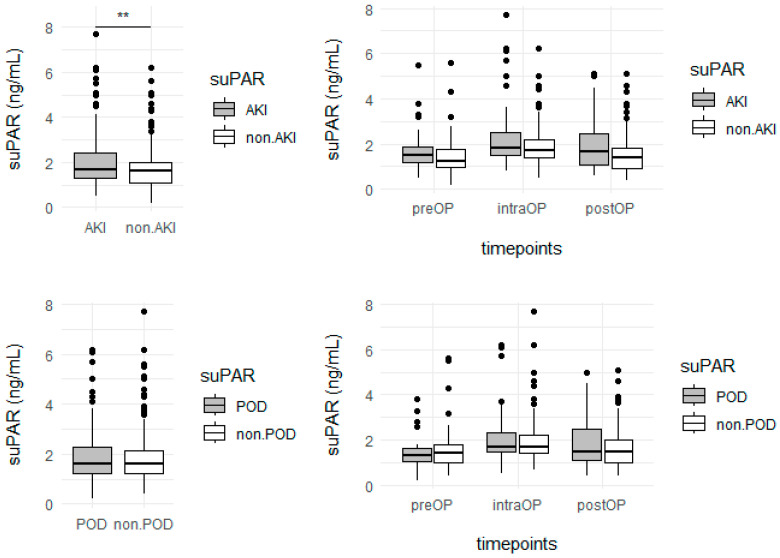
Comparison of suPAR plasma levels in patients with and without AKI or POD. **Top left**: Overall comparison of suPAR levels between patients with acute kidney injury (AKI) and without AKI. suPAR levels were significantly higher in the AKI group (*p* < 0.01). **Top right**: Time course of suPAR levels (preoperative, postoperative, and on the day of surgery) in patients with and without AKI. No significant differences were observed at individual time points. **Bottom left**: Overall comparison of suPAR levels between patients with postoperative delirium (POD) and without POD. No significant difference was observed. **Bottom right**: Time course of suPAR levels in patients with and without POD. No significant differences were observed at any time point. Abbreviations: preOP = preoperative; postOP = postoperative; intra = intraoperative. ** = *p* < 0.01.

Overall, 25 (31.6%) patients suffered from AKI stage 1 or 2, while no patient developed AKI stage 3. Neither baseline renal parameters were elevated in these patients (creatinine: 0.9 [0.8–1.0] mg/dL; urea: 30.5 [27.5–41.25] mg/dL) nor were CPB and aortic cross-clamp (ACC) time prolonged (CPB: 86 min [78 min–111 min]; ACC: 59 min [50 min–79 min]). SuPAR levels differed significantly between patients with or without kidney damage (no AKI: 1.6 [1.1–2.0], AKI: 1.7 [1.3–2.4], *p* < 0.001; see Supplemental Figure S1 and Supplemental Table S2 for a detailed overview of all time points; pre-, intra- and postoperative suPAR kinetics are illustrated in Figure 3). There was no statistical significance between the occurrence of AKI in patients treated with miECC or cCPB (Figure 2A, Table 1). Multivariate regression analysis showed that, in addition to the type of CPB, AKI was an influencing factor on suPAR (−0.45 ± 0.1; *p* < 0.001). To exclude the effect of CPB or ACC duration, we performed an analysis at T3, which revealed no significant difference between patients with and without AKI (Table 3).

Urea decreased significantly on day 1 after surgery and remained significantly lower as compared to the baseline value. However, the amount of change is without clinical relevance (Supplemental Table S2). Creatinine decreased significantly during surgery and then increased to its previous level (except for patients with AKI), whereas GFR increased significantly during surgery and remained at a higher level close to significance. The average pre-, intra-, and postoperative plasma concentration of suPAR showed no correlation with the renal parameters creatinine, urea, and GFR. However, in addition to the significant group difference in the occurrence of AKI, regression analysis revealed a 37% increase in the likelihood of AKI with each one-unit increase in suPAR (odds ratio 1.37 [1.14–1.67]; *p* < 0.001). The ROC analysis of postoperative suPAR revealed only a low predictive capacity for AKI, while intraoperative suPAR levels failed to predict AKI (AUC ROC suPAR: T3 and T4: 0.560; T5 and T6: 0.613).

#### 3.3.2. Postoperative Delirium

Overall, 19 (24.1%) patients developed POD, while two of those had delirium already prior to surgery. SuPAR plasma levels did not differ between patients with and without POD (Figure 2B). suPAR showed limited capacity to predict POD (AUC ROC suPAR: T3 and T4: 0.521; T5 and T6: 0.532). The suPAR kinetics (pre-, intra- and postoperative) are illustrated in Figure 3.

#### 3.3.3. Infectious Complications

No patient developed sepsis, catheter-related bloodstream infections, or urogenital infection. Pneumonia was the most common infectious disease, with two (2.5%) cases. In these patients, suPAR plasma levels in general were significantly increased compared to patients without pneumonia (peak: 3.5 ng/mL at T6 in both patients). Patients with pneumonia were in need of prolonged invasive ventilation (1.3 days compared to 0.8 in the case of no pneumonia). Given that only two patients developed pneumonia, we refrained from calculating the AUC for this outcome. Data on suPAR and the other inflammatory parameters are shown in Supplemental Table S3.

SuPAR plasma levels neither correlated with CRP nor with leukocytes count or PCT, independently of the type of CPB (only postoperative values of cCPB: leukocytes count: correlation coefficient −0.06; *p* = 0.72; CRP: correlation coefficient 0.02; *p* = 0.92; PCT: correlation coefficient −0.10; *p* = 0.58; only postoperative values of MiECC: leukocytes count: correlation coefficient −0.03; *p* = 0.86; CRP: correlation coefficient −0.28; *p* = 0.08; PCT: correlation coefficient 0.03; *p* = 0.88, Supplemental Figure S2). 

## 4. Discussion

This study presents four key findings. First, it demonstrates that the use of miECC in cardiac surgery is associated with significantly lower perioperative suPAR levels compared to cCPB. SuPAR levels increased during surgery, with significantly higher intraoperative values observed in the cCPB group. Despite this, postoperative suPAR levels decreased similarly in both groups. Second, although suPAR levels were significantly higher in patients who developed AKI, their predictive value for AKI was limited. Third, suPAR did not predict POD. Fourth, suPAR was elevated in both patients who developed pneumonia.

The primary objective of this study is to demonstrate that miECC reduces suPAR levels, implying its role in reducing the systemic inflammatory response. The observed increase in suPAR levels during CPB supports previous studies showing that cCPB exacerbates systemic inflammation due to blood contact with artificial surfaces, ischemia-reperfusion injury, and surgical trauma, all of which trigger cytokine release and immune activation [9]. The lower suPAR levels in the miECC group support the hypothesis that minimized circuits reduce the inflammatory burden, as suggested by Ranucci et al., as well as studies by Angelini et al. and Anastasiadis et al. [2,21,22], which further support the anti-inflammatory effects of MiECC in cardiac surgery [20,23]. Studies from the early 2000s found that MiECC was associated with decreased levels of C-reactive protein, interleukin (IL)-6, tumor necrosis factor-alpha, and other cytokines [24,25,26,27,28]. This is primarily explained by the reduced foreign surface area in miECC systems compared to cCPB, resulting in reduced complement activation and subsequent neutrophil activation. Neutrophils play a pivotal role in reacting to foreign surfaces by releasing neutrophil extracellular traps, which interact with the inflammatory and coagulation systems, activating platelets, macrophages, and other immune cells [29,30,31]. This process leads to the release of interleukins, particularly IL-6 and IL-8, strong pro-inflammatory cytokines. suPAR is a part of this cascade, reflecting systemic immune activation and modulating inflammatory responses by interacting with uPAR signaling pathways. As a soluble form of uPAR, suPAR is shed from the cell surface in response to inflammation and immune activation, playing a crucial role in immune cell recruitment, extracellular matrix remodeling, and fibrinolysis.

An important limitation of our study is the potential confounding effect of pre-existing cardiovascular disease on suPAR plasma levels. Patients undergoing cardiac surgery frequently present with a history of myocardial infarction or cerebrovascular events, both of which are associated with chronic systemic inflammation. While we excluded patients with acute stroke, a myocardial infarction within 90 days was present in patients with cCPB. To further assess the influence of recent myocardial infarction, we analyzed preoperative TNI plasma levels, which were significantly higher in cCPB patients. As TNI levels correlated with suPAR, this suggests that myocardial injury may have contributed to elevated suPAR concentrations and could act as a relevant confounder. However, it must be emphasized that atherosclerosis, commonly present in all patients undergoing cardiac surgery, is a chronic inflammatory condition known to elevate suPAR levels, irrespective of recent ischemic events. suPAR has been proposed as a prognostic biomarker in patients with myocardial ischemia for this reason [32,33]. Given the prevalence of atherosclerotic disease in this patient population, complete elimination of this confounding factor is not feasible. Nevertheless, we acknowledge this limitation and have incorporated it into the interpretation of our findings.

Next, the duration of CPB may have influenced the observed differences between MiECC and cCPB, as the duration of cCPB—unlike ACC—was significantly longer compared to MiECC. While T2 reached statistical significance, a similar difference was observed at T3, which represents a time point independent of CPB or ACC duration. Given the visible trend toward higher suPAR levels at T3 and the already significantly elevated levels at T2 in the CPB group, the lack of statistical significance at T3 is most likely attributable to the limited number of included patients.

The secondary aim of this study was to assess whether the decrease in suPAR plasma concentration with miECC is associated with adverse patient outcomes. The results showed that while suPAR was associated with renal impairment, there was no statistically significant difference in AKI occurrence between patients treated with miECC and those treated with cCPB. This suggests that despite the reduction of suPAR levels with MiECC, suPAR still reflects renal damage. However, this study revealed only a moderate predictive capacity of suPAR for AKI detection. This may be due to the short observation period, although the first 24 h after surgery and CPB use are critical for these patients. Therefore, early prediction of AKI remains highly relevant during this period. Rasmussen et al. used this approach and reported that suPAR was a predictor of AKI after cardiac surgery both preoperatively and within the first 24 h [8]. Specifically, the risk for AKI (KDIGO 1) was 50% higher if suPAR plasma levels were doubled. Mossanen et al. supported these findings, also with low predictive performance, comparable to our study (AUC ROC 0.6 vs. 0.647 vs. 0.613) [11]. Furthermore, the duration of CPB must be considered when analyzing the relationship between suPAR and AKI. Although prolonged CPB duration correlated with elevated suPAR levels, it was not associated with the occurrence of AKI in our study. Additionally, the increase in suPAR levels observed at T3, independent of CPB or ACC duration, did not differ between patients with and without AKI. This might be explained by the multifactorial nature of AKI, in which suPAR is not the primary driving factor.

On the one hand, this is surprising, as suPAR is pathophysiologically linked to renal repair mechanisms, but it might be explained by the complex factors influencing AKI. While systemic inflammation plays a significant role in renal damage during cardiac surgery, other factors such as sex, intraoperative hypotension, and pre-existing chronic renal disease can also contribute to renal failure [34,35,36]. Furthermore, suPAR is also released by extrarenal tissues, such as myeloid cells in the bone marrow [37,38]; however, the role of extrarenal sources of suPAR during cardiac surgery remains unclear. On the other hand, this is not particularly unusual, as even well-established biomarkers such as creatinine have limited predictive value for early AKI, given that they primarily reflect reduced GFR as a surrogate marker [39]. Therefore, the early rise in suPAR may still hold promise in multi-marker strategies. Combining suPAR with other established early biomarkers (e.g., NGAL, KIM-1, or cystatin C), clinical parameters, and advanced predictive modeling techniques may improve overall predictive performance. In particular, artificial intelligence and deep learning models could enhance AKI prediction by analyzing the temporal patterns of multiple biomarkers [39,40]. The role of inflammatory biomarkers such as suPAR will be further explored in future studies.

The lack of a significant association between suPAR and POD contrasts with studies linking systemic inflammation to neurocognitive dysfunction after surgery [41]. It is well known that during CPB, a complex inflammatory activation is induced, prompting immune cells, primarily monocytes and neutrophil granulocytes, to secrete proinflammatory cytokines including IL-1β, TNF-α, and IL-6. These cytokines can cross the blood-brain barrier and either directly affect cerebral immune cells like microglia or increase barrier permeability. Endothelial activation plays a crucial role by upregulating adhesion molecule expression, facilitating leukocyte transmigration into brain tissue, and amplifying local inflammation [42,43,44]. This inflammatory cascade may result in persistent neuroinflammation, potentially increasing the risk of postoperative neurocognitive dysfunction. While inflammatory markers such as CRP and interleukins have been associated with POD [45], our data suggest that suPAR alone may not be a reliable biomarker for this outcome. This is surprising, since suPAR has been associated with endothelial damage in the kidney and other organs and with neurocognitive impairment [46,47,48]. The small number of included patients and the variability of data might explain this finding. For this reason, this warrants further investigation into the role of suPAR in neuroinflammation.

Although our study identified significantly elevated suPAR levels in patients with pneumonia [9], the small sample size of infected patients limits the generalizability of this finding. Nevertheless, since prior studies have highlighted suPAR as a potential biomarker for sepsis and infection, it could be an interesting target for future research. Therefore, its clinical utility in predicting postoperative infection requires validation in a larger cohort of patients with infectious complications. 

Several limitations must be considered when interpreting our findings. First, our study cohort was relatively small, limiting statistical power, particularly for subgroup analyses such as pneumonia. Nevertheless, this study yielded important findings that can inform sample size calculations for future research, in line with its exploratory nature. These results support further investigation into suPAR release during cardiopulmonary bypass and underscore the need for larger, prospectively designed studies with more comprehensive baseline phenotyping. Second, this was a secondary analysis of an observational study, and unmeasured confounders may have influenced the results. For instance, only white Caucasians were included in this study. The SAfrEIC study showed that suPAR levels were higher in black compared to white South African males. Further, it has been described that women have a 10% higher plasma level of suPAR [49,50]. Further, the lack of randomization between the miECC and cCPB groups may have introduced selection bias, as the choice of CPB type was based on clinical judgment. This limits the ability to draw causal conclusions regarding the effect of the perfusion technique on biomarker levels. Third, the lack of randomization and the absence of predefined criteria for assigning patients to concurrent studies may have introduced selection bias, despite the strict application of inclusion and exclusion criteria. This limits the ability to draw causal conclusions regarding the effects of CPB type. Fourth, while we observed an association between suPAR levels and AKI, the biomarker’s predictive value was modest, suggesting that additional markers or a multimodal approach may be necessary for accurate AKI risk stratification. Finally, this study focused on elective CABG surgery, and the findings may not be generalizable to other cardiac procedures.

## 5. Conclusions

This study provides evidence that miECC is associated with lower intraoperative suPAR levels, suggesting a reduced inflammatory response compared to cCPB. While suPAR levels were significantly higher in patients with AKI, their predictive value for AKI remains limited. Furthermore, suPAR did not predict POD but was elevated in patients with pneumonia. These findings underscore the potential role of suPAR as an inflammatory biomarker in cardiac surgery, but further studies with larger cohorts and external validation are needed to clarify its clinical utility.

## Figures and Tables

**Table 1:** Basic characteristics. Data are demonstrated as median [interquartile range] or percentage. Significant differences are demonstrated with an asterisk and written in bold (* = *p* < 0.05; ** = *p* < 0.01). Abbreviations: ACC = Aortic cross clamp; CPB = Cardiopulmonary bypass.

Parameters	All Patients (n = 79)	miECC (n = 41)	cCPB (n = 38)	*p* Value
Age (years)	64 [57–71]	60 [56–71]	68 [62–73]	0.06
Male sex, n (%)	69 (87.3)	37 (90.2)	32 (84.2)	0.51
BMI (kg/m^2^)	28.7 [25.9–32.6]	29.1 [27.7–32.0]	28.4 [25.0–33.0]	0.48
EuroSCORE	1.09 [0.78–1.41]	1.07 [0.79–1.29]	1.13 [0.77–1.55]	0.51
**Pre-existing Diseases**, n (%)				
Angina pectoris	53 (68.8)	29 (72.5)	24 (64.9)	0.62
Arterial hypertension	68 (87.2)	37 (90.2)	31 (83.8)	0.50
Acute myocardial infarction	20 (25.6)	9 (22.0)	11 (29.7)	0.45
Myocardial infarction within the last 90 days prior to surgery	18 (23.4)	4 (10.0)	14 (37.8)	0.006 (**)
Concurrent valvular disease	16 (20.5)	11 (26.8)	5 (13.5)	0.17
Stroke	10 (12.8)	2 (4.9)	8 (21.6)	0.041 (*)
Diabetes	32 (40.5)	16 (39.0)	16 (43.2)	0.82
Chronic obstructive pulmonary disease (>1)	10 (12.8)	5 (12.2)	5 (13.5)	0.88
Smoker	51 (65.4)	26 (63.4)	25 (67.6)	0.81
Alcohol abuse	1 (1.3)	1 (2.4)	0 (0)	1.00
**Outcome,** n (%)				
Postoperative delirium	19 (24.1)	9 (22.0)	10 (26.3)	0.79
Acute kidney disease (KDIGO > 1)	25 (31.6)	15 (36.6)	10 (26.3)	0.35
Pneumonia	2 (2.5%)	1 (2.4%)	1 (2.6%)	1.00
**Process times**				
Duration of anesthesia (min)	202 [177.0–232.0]	193.5 [176.0–224.0]	214.6 [185.0–247.5]	0.12
Duration of CPB (min)	82.5 [72–107.5]	78.0 [71.5–91.5]	90.0 [76.0–114.0]	**0.049** (*)
Duration of ACC (min)	57 [47–77]	56 [47–66.5]	60 [48–85]	0.19
Duration of invasive ventilation (h)	12.4 [10.4–18.2]	12.8 [9.5–18.0]	13.9 [11.0–19.1]	0.30

**Table 2:** Overview of the suPAR quantification. Measurements are given as median [interquartile range], *p*-value is given for comparison, MiECC vs. cCBP. Abbreviations: cCPB = conventional cardiopulmonary bypass; miECC = Minimized extracorporeal circulation; suPAR = Soluble urokinase plasminogen activator receptor. * = *p* < 0.05; ** = *p* < 0.01.

	n	suPAR (ng/mL) All Patients	n	suPAR (ng/mL) miECC	n	suPAR (ng/mL) cCPB	Two-Groups *p*-Value (WILCOX)
T1 (preoperative)	79	1.4 [1–1.8]	41	1.3 [1–1.6]	38	1.4 [1.03–2.12	0.29
T2 (15’ CPB)]	76	1.8 [1.4–2.2]	41	1.7 [1.4–2]	35	1.9 [1.55–2.5]	0.049 (*)
T3 (60’ CPB)	78	1.8 [1.52–2.28]	40	1.7 [1.58–2]	38	2 [1.52–2.58]	0.12
T4 (15’ after end of CPB)	78	1.7 [1.3–2.28]	40	1.5 [1.3–2.1]	38	1.9 [1.4–2.58]	0.09
T5 (120’ after end of CPB)	77	1.6 [1.2–2.3]	41	1.5 [1.1–1.7]	36	1.9 [1.3–2.96]	0.008 (**)
T6 (1st day postoperative)	73	1.2 [0.9–1.7]	40	1.25 [0.88–1.63]	33	1.2 [1–1.9]	0.44
All operative time points (T2–T4)	232	1.7 [1.4–2.22]	121	1.7 [1.4–2]	111	1.9 [1.44–2.55]	0.003 (**)
All postop. time points (T5–T6)	150	1.5 [1–2.08]	81	1.4 [0.9–1.7]	69	1.6 [1.1–2.6]	0.02 (*)

**Table 3:** Overview of the suPAR quantification. Wilcoxon test. *P*-value is given for 2-group comparisons according to column labels. Results for two-factor ANOVA analysis are given in the remaining rows. Abbreviations: cCPB = conventional cardiopulmonary bypass; MiECC = Minimized extracorporeal circulation; suPAR = Soluble urokinase plasminogen activator receptor. * = *p* < 0.05; ** = *p* < 0.01; *** = *p* < 0.001.

	AKI/no AKI	POD/noPOD	miECC/cCBP
Wilcox-Test	0.003 (**)	0.67	<0.001 (***)
ANOVA pVal	0.001 (**)	0.03 (*)	<0.001 (***)
T1/preoperative	0.98	1.00	1.00
T2/operative	0.98	1.00	0.99
T3/operative	1.00	1.00	0.78
T4/operative	0.92	1.00	0.48
T5/postoperative	0.82	1.00	0.11
T6/postoperative	0.83	1.00	1.00
ANOVA pVal	**<0.001** (***)	0.02 (*)	**<0.001** (***)
preoperative	0.79	1.00	0.98
operative	0.26	1.00	0.03 (*)
postOP	0.13	0.88	0.16

## Data Availability

The datasets used and/or analyzed during the current study are available from the corresponding author on reasonable request.

## Supplementary Materials

The following supporting information can be downloaded at https://www.mdpi.com/article/10.3390/jcm14145020/s1: Table S1: Details on the used CPB materials. Abbreviations: CPB = Cardiopulmonary bypass, cCPB = Conventional cardiopulmonary bypass, MiECC = Minimized extracorporeal cardiopulmonary bypass; Table S2: Overview of the suPAR quantification in dependence on the occurrence of AKI. Measurements are given as median [interquartile range]. Abbreviations: cCPB = conventional cardiopulmonary bypass; miECC = Minimized extracorporeal circulation; suPAR = Soluble urokinase plasminogen activator receptor; Table S3: Overview of the inflammatory parameters at different time points. Values are given as median [IQR]. Abbreviations: CRP = C-reactive protein; GFR = glomerular filtration rate; PCT = Procalcitonin; IQR = interquartile range; N.A. = Not available; Figure S1: Boxplots showing the time course of suPAR in dependence on the occurrence of AKI. Abbreviations: cCPB = conventional cardiopulmonary bypass; miECC = Minimized extracorporeal circulation; suPAR = Soluble urokinase plasminogen activator receptor; AKI = acute kidney injury; Figure S2: Boxplots showing the time course of the inflammatory parameters and suPAR. Abbreviations: CRP = C-reactive protein. PCT = Procalcitonin, preOP = preoperative; postOP = postoperative; OP = intraoperative, suPAR = soluble urokinase plasminogen activator receptor.
