# Supplementary material for: Soluble Urokinase Plasminogen Activator Receptor (suPAR) Plasma Concentration Is Reduced Using Minimized Extracorporeal Circulation: Results of a Secondary Analysis of a Prospective Observational Study
Source: J Clin Med. 2025 Jul 16;14(14):5020. doi: 10.3390/jcm14145020 (PMC12296034; doi:10.3390/jcm14145020)
Supplement: Supplementary file 1 [file jcm-14-05020-s001.zip › jcm-3676123-supplementary.pdf]

Supplemental Table S1. Details on the used CPB materials. Abbreviations: CPB = Cardiopulmonary bypass, cCPB = Conventional cardiopulmonary bypass, MiECC = Minimized extracorporeal cardiopulmonary bypass.

	**MiECC**	**cCPB**
**Tubing**	3/8 Inch, coated (phosphorylcholin)	3/8 Inch, coated (phosphorylcholin)
**Pump**	Centrifugal pump	Roller pump
**Cardiotomy reservoir**	No	Yes
**Venous bubble trap**	Yes	No
**Venous reservoir**	No	Yes
**Cardiotomy suction**	No	Yes
**Cell saver**	Yes	Optional (no standard)
**Priming volume**	1050 ml (active priming: 800 ml)	1300 ml
**Composition**	Balanced full electrolyte solution 1000ml, human albumin 20% 50ml, unfractioned Heparin 10000IE Protamin	Balanced full electrolyte solution 1000ml, human albumin 20% 50ml, unfractioned Heparin 10000IE, mannitol 15% 250ml
**Cardioplegia**	Calafiore	Buckberg + Calafiore (no Standard)
**Heart-Lung Machine**	Livanova S5	Livanova S5

Supplemental Table S2. Overview of the suPAR quantification in dependence on the occurrence of AKI. Measurements are given as median [interquartile range]. *Abbreviations: cCPB = conventional cardiopulmonary bypass; miECC = Minimized extracorporeal circulation; suPAR = Soluble urokinase plasminogen activator receptor.*

	suPAR (ng/mL) All patients	***suPAR (ng/mL) AKI***	***suPAR (ng/mL) non-AKI***
T1 (preoperative)	1.37 [1-1.8]	1.5 [1.2-1.9]	1.25 [0.98-1.78]
T2 (15´ CPB)]	1.8 [1.4-2.2]	1.8 [1.5-2.5]	1.7 [1.4-2.1]
T3 (60´ CPB)	1.8 [1.52-2.28]	1.8 [1.6-2.5]	1.8 [1.5-2.1]
T4 (15´ after end of CPB)	1.7 [1.3-2.28]	1.7 [1.5-2.4]	1.6 [1.3-2.2]
T5 (120´ after end of CPB)	1.6 [1.2-2.3]	1.8 [1.37-3.1]	1.5 [1.2-2.1]
T6 (1st day postoperative)	1.2 [0.9-1.7]	1.5 [1-2.4]	1.2 [0.9-1.7]
All operative time points (T2-T4)	1.7 [1.4-2.22]	1.8 [1.5-2.5]	1.7 [1.4-2.2]
All postop. time points (T5-T6)	1.5 [1-2.08]	1.65 [1.1-2.47]	1.4 [0.92-1.8]

Supplemental Table S3. Overview of the inflammatory parameters at different time points. Values are given as median [IQR]. Abbreviations: CRP = C-reactive protein; GFR = glomerular filtration rate; PCT = Procalcitonin; IQR = interquartile range; N.A. = Not available.

		**Preoperative (T1)**	**Intraoperative (T2-4)**	**Postoperative (T5-6)**
Inflammation parameters				
**Leukocytes (number/µL)**	***All patients***	7.6 [6.7-9.07]	12 [7.9-14.7]	12.2 [9.9-14.4]
	miECC	7.4 [6.7-9.37]	12.7 [8.9-15.7]	12.3 [10.3-14.3]
	cCPB	7.85 [6.93-8.97]	10.65 [7.4-13.88]	12.15 [9.33-14.43]
**CRP (mg/mL)**	***All patients***	3.53 [0.7-10.57]	3.8 [0.68-8.21]	92.71 [72.71-116.56]
	miECC	2.97 [0.78-9.97]	4.16 [0.56-8.55]	96.91 [72.3-115.77]
	cCPB	5.18 [0.7-12.54]	3.64 [0.79-7.21]	87.86 [75.89-130.07]
**PCT (µg/L)**	***All patients***	4.2 [3.9-4.45]	N.A.	4.55 [4.3-4.7]
	miECC	4.3 [3.9-4.4	N.A.	4.4 [4.28-4.7]
	cCPB	4.2 [3.9-4.5]	N.A.	4.6 [4.5-4.8]
Kidney parameters				
**GFR (ml/min)**	***All patients***	89.75 [78.4-107.25]	102 [87.5-118]	89.5 [77.5-107]
	miECC	84.9 [78.17-106.1]	102 [87.25-117.75]	88.7 [72-107]
	cCPB	98.75 [81.8-116.93]	102 [88-119]	91 [79-107.25]
**Urea (mg/dL)**	***All patients***	30 [26-37]	26 [22-30]	27 [22.75-32]
	miECC	34.5 [28-39.5]	27 [23-31.25]	28 [22.25-32]
	cCPB	28 [24.25-32.75]	25.5 [21-27.75]	27 [23.25-31.5]
**Creatinine (mg/dL)**	***All patients***	0.9 [0.7-1]	0.8 [0.7-0.9]	0.9 [0.8-1]
	miECC	0.95 [0.8-1]	0.8 [0.7-0.9]	0.9 [0.8-1]
	cCPB	0.8 [0.7-0.9]	0.8 [0.7-0.9]	0.85 [0.8-0.98]

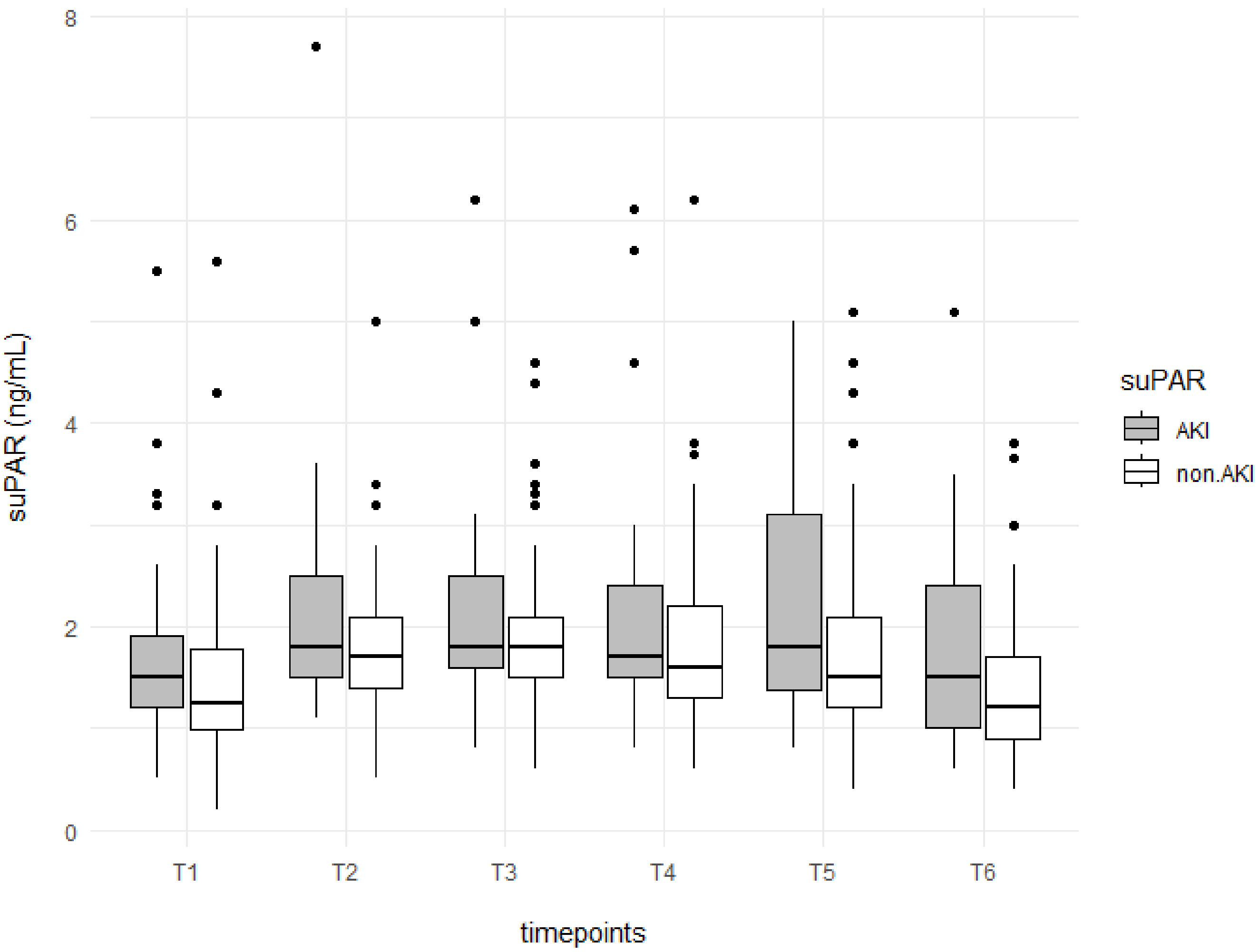

Supplemental Figure S1. Boxplots showing the time course of suPAR in dependence on the occurrence of AKI. Abbreviations: cCPB = conventional cardiopulmonary bypass; miECC = Minimized extracorporeal circulation; suPAR = Soluble urokinase plasminogen activator receptor; AKI = acute kidney injury.

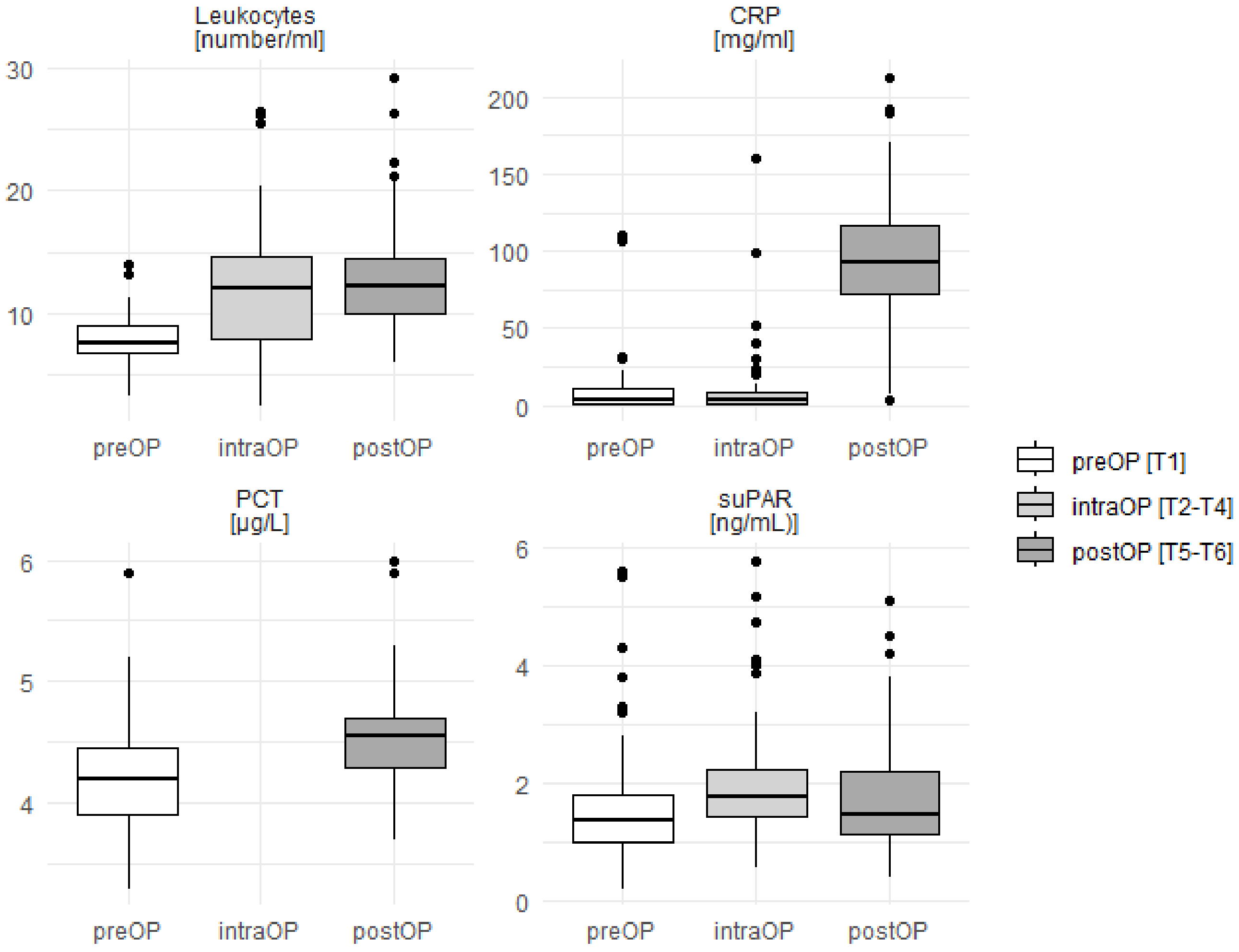

Supplemental Figure S2. Boxplots showing the time course of the inflammatory parameters and suPAR. *Abbreviations: CRP = C-reactive protein. PCT = Procalcitonin, preOP = preoperative; postOP = postoperative; OP = intraoperative, suPAR = soluble urokinase plasminogen activator receptor.*
